# Challenges to Using an Electronic Personal Health Record by a Low-Income Elderly Population

**DOI:** 10.2196/jmir.1256

**Published:** 2009-10-27

**Authors:** Eung-Hun Kim, Anna Stolyar, William B Lober, Anne L Herbaugh, Sally E Shinstrom, Brenda K Zierler, Cheong B Soh, Yongmin Kim

**Affiliations:** ^4^School of Electrical & Electronic EngineeringNanyang Technological UniversitySingapore; ^3^Behavioral Nursing and Health SystemsUniversity of WashingtonSeattleWAUSA; ^2^Medical Education and Biomedical InformaticsUniversity of WashingtonSeattleWAUSA; ^1^BioengineeringUniversity of WashingtonSeattleWAUSA

**Keywords:** Personal health record (PHR), personally controlled health record (PCHR), elderly populations, low-income populations, Web-based, Internet

## Abstract

**Background:**

Electronic personal health records (PHRs) are increasingly recognized and used as a tool to address various challenges stemming from the scattered and incompatible personal health information that exists in the contemporary US health care system. Although activity around PHR development and deployment has increased in recent years, little has been reported regarding the use and utility of PHRs among low-income and/or elderly populations.

**Objective:**

The aim was to assess the use and utility of PHRs in a low-income, elderly population.

**Methods:**

We deployed a Web-based, institution-neutral PHR system, the Personal Health Information Management System (PHIMS), in a federally funded housing facility for low-income and elderly residents. We assessed use and user satisfaction through system logs, questionnaire surveys, and user group meetings.

**Results:**

Over the 33-month study period, 70 residents participated; this number was reduced to 44 by the end of the study. Although the PHIMS was available for free and personal assistance and computers with Internet connection were provided without any cost to residents, only 13% (44/330) of the eligible residents used the system, and system usage was limited. Almost one half of the users (47%, 33/70) used the PHIMS only on a single day. Use was also highly correlated with the availability of in-person assistance; 77% of user activities occurred while the assistance was available. Residents’ ability to use the PHR system was limited by poor computer and Internet skills, technophobia, low health literacy, and limited physical/cognitive abilities. Among the 44 PHIMS users, 14 (32%) responded to the questionnaire. In this selected subgroup of survey participants, the majority (82%, 9/11) used the PHIMS three times or more and reported that it improved the quality of overall health care they received.

**Conclusions:**

Our findings suggest that those who can benefit the most from a PHR system may be the least able to use it. Disparities in access to and use of computers, the Internet, and PHRs may exacerbate health care inequality in the future.

## Introduction

Health care systems around the world are facing various challenges. In the United States in particular, the health care system is considered expensive, fragmented, unsafe, and unequal [1], although many innovations in medical diagnosis and treatment have been pioneered and made clinically available [2]. Over the past several years, health information technologies, such as electronic health records (EHRs) and personal health records (PHRs), have emerged and have been promoted by experts, industry, and government as an effective tool to address the inefficiencies and disadvantages of the current health care system [3-12]. The EHR and PHR systems hold the promise of improving the quality of health care services by improving communication within and across the health care system, reducing medical errors and waste of health care resources, and simplifying the complexity inherent in redundant information from fragmented sources.

The EHR refers to a computerized health history of an individual that can be viewed as a collection of electronic medical records and other health-related information to be used and viewed primarily by care providers [5,13]. On the other hand, the predominant model of PHRs is an electronic repository of personal health information to be managed and accessed by patients and others authorized by patients [5,6]. Although the EHR and PHR have different end-user groups, they contain similar information. Ideally, they should be interoperable. In the past few years, adoption of EHRs has been encouraged, whereas PHRs have not received the same level of attention. However, as Tang and Lansky [14] and Ball et al [15] discussed, the EHR alone may lack the ability to sufficiently motivate and engage patients to take a more active role in managing their own health, a condition found critical for improving care quality and efficiency [16]. Therefore, PHRs have been recognized as a means of patient engagement. An EHR-coupled PHR, which is often referred as a patient-accessible EHR [13] or tethered PHR [5], has been increasingly offered in the United States to patients as an institution-specific (limited to a certain organization) Internet portal by some large health care organizations (eg, Kaiser Permanente, Veterans Health Administration, Group Health Cooperative, CareGroup Health Care System, and Palo Alto Medical Foundation).

Enabled by information and communication technology (ICT) and spurred by trends of moving toward patient-centered care, the public interest in accessing and managing personal health information has been growing [17]. Relatively new applications, such as Microsoft HealthVault and Google Health, make a stand-alone PHR available to anyone with Internet access. In spite of the widespread interest and availability of PHRs, their use and utility among the primary users (ie, patients themselves) is not well documented or analyzed [18,19]. Particularly, little work has been done for the elderly and low-income population. Due to the high incidence and prevalence of chronic conditions that generally require frequent monitoring and interventions, elderly people would benefit more because the PHR system could enable more coordinated and cost-effective communication and health care delivery.

**Figure 1:**
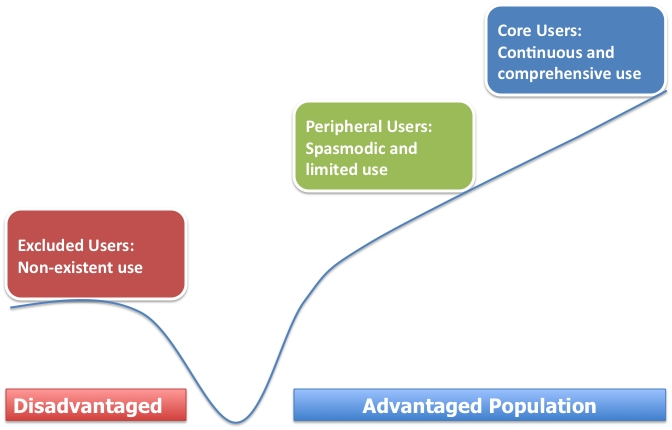
Digital divide (model adopted from Murdock [22]: three groups of ICT users were described in terms of levels of access and use; blue line represents the conceptual valley and barriers of the digital divide)

Compared with younger and/or more affluent counterparts, the elderly with low income are likely to be disadvantaged in using PHRs due to the disparities in accessing and using ICT, referred as the “digital divide.” The digital divide is defined as the gap that exists between individuals, groups, or communities in terms of the availability of ICT and the ability to use these technologies effectively [20]. Although the availability of Internet access has been steadily increasing, only 40% of low-income families (those with less than US$20,000 household income) have Internet access compared to 73% of the overall US population according to a survey conducted in 2008 [21]. The survey also found that although Internet use among adults aged 50 or older has shown the highest growth rates, only 35% of this population have Internet access. Therefore, the low-income elderly are more likely to be on the underprivileged side of the digital divide, and many would be classified as excluded users based on Murdock’s categorization as illustrated in Figure 1 [22]. This divide was observed in a study using a tethered PHR: healthier, socioeconomically advantaged, health-minded, and younger individuals were more likely to use the portal [23]. Hsu et al [24] also found the widening divide over time (from 1999 to 2002) in the adoption of PHRs and related applications between the low socioeconomic group and its counterpart.

In this paper, we present a 33-month study of an institution-neutral, “stand-alone” PHR used by low-income elderly residents living in a subsidized housing facility. This PHR system, the Personal Health Information Management System (PHIMS), allowed users to enter and manage their health information with the help of student nurses. In this paper, we present the results of this exploratory study, the utility and use of PHIMS in this socioeconomically disadvantaged population, and user satisfaction.

## Methods

The PHIMS is an institution-neutral (not bound to any organization), individually controlled, Web-based repository of personal health information [25]. It allows users to enter, update, or delete structured information in nine different categorizes. Each category has multiple information elements. For example, under medications, one can record dosage, effectiveness, prescribing doctor’s name, and reasons for taking/stopping each medication. Many categories have free-text boxes where any additional information a user wants to record can be entered. Some of these text boxes are used to enter questions or topics a user wishes to discuss with providers. The PHIMS provides summary pages that list all the information a user has entered into the system. He or she can share a hardcopy and/or electronic copy with health care providers or family members.

The PHIMS was deployed in a housing complex located in Everett, WA, USA, which serves approximately 500 households. Most residents have a household income below 100% of the federal poverty line, although the eligibility for residency is below 250%. The majority of residents in the complex are the elderly (ie, age 65 or over), who have a high prevalence of multiple chronic illnesses. The PHIMS was initially deployed in December 2004 in one apartment that serves approximately 180 residents. In May 2006, a second location with around 150 residents was added. Socioeconomic status and ages of the residents in the second location were similar to those in the first apartment, except that about 30% (45/150) of them were immigrants whose primary language was Russian.

The PHIMS was made available to all adult residents (most residents were adults) from December 2004 (May 2006 for the second location) to August 2007. In 2004, approximately 80% (145/180) of residents did not have Internet access. Consequently, a computer room equipped with six PCs with a broadband Internet connection and a printer was set up for the residents. When the deployment was expanded in 2006, the second location already had a computer room with four Internet-linked PCs and two printers. Two graduate nursing students visited the complex once a week (mostly Thursdays from 10:00 am to 2:00 pm) to help the residents create and manage (enter, update, delete, or print) their personal health information. One housing staff member (social worker) occasionally helped the residents as well. For Russian-speaking residents, an interpreter service was also made available.

We conducted various informational sessions to explain what the PHIMS was and to demonstrate how to use it [25,26]. The study was approved by the Institutional Review Board at the University of Washington. All participating residents (PHIMS users) accepted the terms of the online consent form. 

System usage (ie, user activities), such as information updates and retrievals, was analyzed from the system logs. The logs recorded the details of user activities, including accessed information category, activity type (eg, enter, update, or delete), and the date, time, and duration of each access.

In August 2007, a (paper) questionnaire was administered to the users of the system to assess overall satisfaction with PHIMS and obtain self-reported comments on their experience.

The questionnaire responses and the system logs were analyzed using MATLAB with Statistics Toolbox (The Mathworks, Inc, Natick, MA, USA). Exploratory descriptive statistics were mostly used to analyze the questionnaire responses and the frequencies and patterns of user activities.

## Results

### Participation

A total of 70 residents participated in the study. Table 1 describes the age and gender distribution of the PHIMS users. The average age of participants was 63.1 years (SD = 15.4 years), which was not significantly different (*P* = .23, Student unpaired *t*_298_ test) from all the residents in the housing complex (mean = 65.8, SD = 15.7 years). Of the 70 participants, 44 (63%) were older than 60 years, and 71% (50/68) were female. The gender of PHIMS users was not significantly different from the resident population (*P* = .27, Fisher exact test). All participants indicated that their primary language was English.

**Table 1:** Age and gender distribution of PHIMS users (N = 70)

		Number (%)
**Age (years)**		
	21-30	2 (2.9)
	31-40	2 (2.9)
	41-50	8 (11.4)
	51-60	14 (20.0)
	61-70	27 (38.6)
	71-80	5 (7.1)
	81-90	7 (10.0)
	91-100	5 (7.1)
**Gender**		
	Male	18 (25.7)
	Female	50 (71.4)
	Not disclosed	2 (2.9)

### System Usage

Three users used the PHIMS for 25, 21, and 17 days each. On the other hand, 33/70 participants (47%) used the PHIMS only on a single day during the study period, as shown in Table 2. If we limit the users to those who had at least 12 months to use the PHIMS (n = 53), more than half (51%, 27/53) accessed the system only one day during their first-year PHIMS use.

The system was most frequently used on Thursdays (67%, 5387/8008), followed by Fridays (14%, 1098/8008), which coincided with the onsite availability of graduate nursing students. Most (77%, 6174/8008) of the system use happened while assistance from graduate nursing students or housing staff was available to the residents. On the other hand, 8% (677/8008) of user activities occurred during off hours when the students or staff were not available (from 5:00 pm to 8:00 am weekdays and weekends).

**Table 2:** Number of discrete days of PHIMS use during the study period for all users (N = 70) and during the first 12 months (N = 53)

Number of Days Used	All Users, No. (%)	Users for the First 12 Months, No. (%)
1	33 (47.1)	27 (50.9)
2	17 (24.3)	11 (20.8)
3	6 (8.6)	3 (5.7)
4	3 (4.3)	4 (7.5)
5	5 (7.1)	4 (7.5)
6+	6 (8.6)^a^	4 (7.5)^b^

^a^6, 9, 17, 21, and 25 discrete days of use.

^b^8, 10, 16, and 17 discrete days of use.

### Survey Responses

In August 2007, only 44/70 PHIMS users were still living in the housing complex. Some had moved out of the building due to changes in their financial status and other reasons, and some had passed away during the study period. Among the 44 PHIMS users, 14 (32%) responded to the questionnaire. A total of 79% (11/14) of the survey respondents said that they entered health information by themselves at least once. Except the three respondents who had used the PHIMS for less than 6 months, 82% (9/11) used the PHIMS three times or more. The average age of the survey participants was 65.5 years (SD = 9.8 years).

Textbox 1 shows a summary of survey responses. Most respondents (12/13, 92%) were satisfied with the PHIMS. All shared their PHIMS records with care providers, family members, and/or friends; 93% (13/14) shared their records with their primary care providers and/or specialists. All the respondents judged that with the PHIMS they were able to provide more health information to the providers. Most respondents (10/11, 91%) found that the PHIMS made their face-to-face meetings with providers efficient and felt more prepared for emergencies and in control of coordinating their care.

**Summary of survey responses (95% confidence intervals are calculated based on the adjusted Wald method)**

- 14/14 (100%; 95% CI = 80.9-100) shared their PHIMS record with
  - primary care provider and/or specialist: 13 (92.9%; 95% CI = 66.5-100)
  - family member: 6 (42.9%; 95% CI = 21.3-67.5)
  - friends: 1 (7.1%; 95% CI = 0-33.5)

- 12/12 (100%; 95% CI = 78.4-100) felt that they were able to provide more health information to their health care provider with PHIMS
- 11/12 (91.7%; 95% CI = 62.5-100) felt that they were more prepared for medical emergencies with PHIMS
- 10/11 (90.9%; 95% CI = 60.1-100) indicated that their face-to-face meeting time with their health care provider was used more efficiently with PHIMS
- 9/11 (81.8%; 95% CI = 51.2-96.0) indicated that PHIMS improved the quality of overall health care they received
- 12/13 (92.3%; 95% CI = 64.6-100) were overall satisfied with the PHIMS system

## Discussion

To our knowledge, this is the first study on stand-alone PHR use involving a homogeneous group of subjects living in a low-income housing facility where the majority of residents were elderly.

### Principal Findings

The results from this study underscore challenges in the deployment and widespread adoption of PHRs by socioeconomically disadvantaged populations. Since all the residents were low income and the majority were elderly, most residents belonged to the disadvantaged group in Figure 1. The digital divide includes a technical divide based on the availability of ICT infrastructure, hardware, and software and a social divide resulting from the skills required to manipulate and utilize technical resources [27]. To help the residents overcome these technical and social divides in PHIMS use, PCs, Internet connection, and assistance from nursing students and housing staff were made available free of charge. In spite of this support, the participation rate in using the PHIMS was not much different from previous studies with tethered PHRs that reported a participation rate from 9.3% to 25% with the general population [23,24,28,29]. During our study period of 33 months, the PHIMS attracted 70 users, and in August 2007, 44 still lived in the residence, about 13% (44/330) of the eligible residents. If only residents whose primary language was English are counted, the PHIMS user group is approximately 15% of the eligible residents (44/285).

Compared with the study by Hsu et al [24], who reported 5.3% of people between 50 and 74 years and 2.8% living in a low socioeconomic neighborhood using PHRs without any help, the PHIMS participation rate of 13% indicates that the infrastructure and assistance helped some residents overcome an initial barrier and fear toward using the PHIMS. If the resources and support had not been provided, participation rates would have been lower. In fact, we found that only 8.6% (6/70) of the participants and 1.5% of the eligible residents (5/330 in August 2007) were able to use PHIMS independently without any assistance. Not surprisingly, this group of independent users were the most frequent PHIMS users. If the PHIMS users were an unbiased sample of the residents, the independent users could account for 8.6% of the residents. However, the PHIMS users were self-selected and a biased sample. Thus, the proportion of independent users among the elderly with low income is closer to 1.5% than to 8.6%.

Overall, system usage was limited. Almost one half of the users used the PHIMS only on a single day. In addition, user activities highly correlated with the availability of assistance. Nearly 80% of the user activities occurred during the time when the graduate nursing students and/or housing staff were present on site. The graduate nursing students provided assistance to the residents for only about 4 hours per week during the academic quarters. However, 63% of the total user activities (5035/8008) coincided with their on-site availability. This high dependency was mainly due to the limited physical and cognitive abilities and technophobia (ie, computer anxiety) of the residents, as we had found in an earlier study [26]. While some residents were enthusiastic about using the PHIMS, others expressed fear over computers and the Internet. Among the PHIMS users, 58% had computer anxiety and were initially afraid of using a computer [26]. Therefore, they needed emotional support to overcome their fear. Low health literacy (29% in [26]) was also an important factor that limited PHIMS use. Some users (at least 5 among 13 survey respondents) who said that they could use the PHIMS by themselves commented that they preferred to use it with a nursing student who could not only help in updating records but also provide explanations for them to understand their health information. Both language and culture were formidable barriers in the digital divide, as Keniston [30] identified. None of the Russian-speaking residents used the PHIMS even though interpreter services were made available to them and many had previously expressed interest during information sessions.

In spite of the fact that the participation rate was low and PHIMS use was infrequent, those users who participated in the survey found PHIMS beneficial. Particularly, the respondents who had shared their PHIMS information with their care providers felt very positive about PHIMS and noted the system’s usefulness. With a printout of their PHIMS summary, they were able to provide health information to providers accurately and quickly, leading to better communication with their care providers. This result is consistent with various studies on the impact on patient-provider relationships and communication of using Web-based, provider-supplied health information systems [31-36].

### Limitations

Potential biases in survey responses should be noted. More than half (57%, 8/14) of survey participants said that they were able to use and update the PHIMS on their own most or all the time, whereas only 3/14 (21%) said that they never used or updated the PHIMS by themselves. This self-use rate is quite different from our earlier study, where almost 80% of participants needed assistance to use and update the PHIMS and more than 60% had low computer literacy [26]. Thus, survey respondents in the current study were more computer literate and self-sufficient users than those users who did not participate in the survey. In addition, 82% (9/11) of survey participants who used the PHIMS for 6 months or more used it three times or more, whereas almost half of users used the PHIMS only once. Therefore, the survey respondents were those who were more active users of the PHIMS, and their responses might have not represented those PHIMS users who have only used the system once. However, we were able to clearly observe that at least a fraction of the population in the study was able to receive the benefits of the PHIMS and reported improvements in their perceived quality of care.

Another aspect to be noted is that the PHIMS is an institutional-neutral, untethered (stand-alone) PHR. It contains only self-reported data, the majority of which were entered with the assistance from graduate nursing students. In tethered, provider-supplied PHRs, the majority of personal health information can be added from multiple existing sources, including the provider’s information systems. Therefore, the adoption rate, utility, and use frequency of specific features with tethered PHRs could be somewhat different from those observed in our study, although we found some similarities as well. In our earlier study [25], we found that the medication information was the most frequently used and updated category in the PHIMS, which is similar to the use of tethered PHRs [9,23]. On the contrary, the lab test was one of the least frequently used information categories in the PHIMS, whereas in tethered PHRs, the lab test was one of the most popular features. This may be due to the fact that in a tethered PHR users check lab test results made available by their provider, whereas in a self-entry PHR users enter test results they have received. Because of the reduced workload in managing personal health information, related to not having to enter data into their record themselves, tethered PHRs might be more easily adopted than untethered PHRs. However, even tethered PHRs may not be able to address root causes that limit the use of PHRs, such as age-related reduced physical and cognitive abilities of the low-income elderly. Thus, we believe that the low-income elderly would face similar challenges found in this study whether using a tethered or untethered PHR.

### Concluding Remarks

In the last several years, momentum has been building toward widespread deployment of health information technology in the United States. Earlier studies on PHRs demonstrated their usefulness in improving the quality of care for patients with chronic illnesses [37], in controlling costs [3], and in reducing health care disparities [38-40]. The accelerated efforts from ICT and health care companies and their partnerships will likely substantially increase the availability and usability of PHRs. Furthermore, US government incentives and support as well as broad-based health care reform initiatives now being discussed will facilitate PHR deployment and use by patients and care providers.

It is widely believed that the elderly would benefit more from PHR use than would younger populations [17,41]. However, our findings suggest that the majority of the low-income elderly would not be in a position to benefit from PHRs due to poor technical skills, technophobia, low health literacy and limited physical/cognitive abilities, leaving only a small fraction who can take advantage of PHRs to the full extent. As a result, PHRs may mainly serve self-proficient, advantaged individuals, which could result in further widening of the inequality in health care. As the next-generation elderly population will be more computer literate than the current generation, PHR use among the elderly will increase in the future. However, their low or reduced physical/cognitive abilities due to aging and low health literacy would limit the PHR use. Therefore, many of the underprivileged in the digital divide would be left behind in receiving the benefits that are enabled by ever-advancing PHR systems and their clinical applications, potentially exacerbating the health care inequality.

## Abbreviations

EHR: electronic health record
ICT: information and communication technology
PC: personal computer
PHIMS: Personal Health Information Management System
PHR: personal health record

## References

[1] Institute of Medicine (2001). Crossing the Quality Chasm: A New Health System for the 21st Century.

[2] Groves Trish (2008). Stronger European medical research. BMJ.

[3] Evans Dwight C, Nichol W Paul, Perlin Jonathan B (2006). Effect of the implementation of an enterprise-wide Electronic Health Record on productivity in the Veterans Health Administration. Health Econ Policy Law.

[4] Linder Jeffrey A, Ma Jun, Bates David W, Middleton Blackford, Stafford Randall S (2007). Electronic health record use and the quality of ambulatory care in the United States. Arch Intern Med.

[5] Tang Paul C, Ash Joan S, Bates David W, Overhage J Marc, Sands Daniel Z (2006). Personal health records: definitions, benefits, and strategies for overcoming barriers to adoption. J Am Med Inform Assoc.

[6] Markle Foundation (2004). Connecting for Health. Achieving Electronic Connectivity in Healthcare: A Preliminary Roadmap from the Nation's Public and Private-Sector Healthcare Leaders.

[7] Yasnoff William A, Humphreys Betsy L, Overhage J Marc, Detmer Don E, Brennan Patricia Flatley, Morris Richard W, Middleton Blackford, Bates David W, Fanning John P (2004). A consensus action agenda for achieving the national health information infrastructure. J Am Med Inform Assoc.

[8] Markle Foundation (2003). Connecting for Health. The Personal Health Working Group: Final Report.

[9] Halamka John D, Mandl Kenneth D, Tang Paul C (2008). Early experiences with personal health records. J Am Med Inform Assoc.

[10] Hillestad Richard, Bigelow James, Bower Anthony, Girosi Federico, Meili Robin, Scoville Richard, Taylor Roger (2005). Can electronic medical record systems transform health care? Potential health benefits, savings, and costs. Health Aff (Millwood).

[11] Ferguson Tom, Frydman Gilles (2004). The first generation of e-patients. BMJ.

[12] Chaudhry Basit, Wang Jerome, Wu Shinyi, Maglione Margaret, Mojica Walter, Roth Elizabeth, Morton Sally C, Shekelle Paul G (2006). Systematic review: impact of health information technology on quality, efficiency, and costs of medical care. Ann Intern Med.

[13] Wiljer David, Urowitz Sara, Apatu Emma, DeLenardo Claudette, Eysenbach Gunther, Harth Tamara, Pai Howard, Leonard Kevin J, Canadian Committee for Patient Accessible Health Records (2008). Patient accessible electronic health records: exploring recommendations for successful implementation strategies. J Med Internet Res.

[14] Tang Paul C, Lansky David (2005). The missing link: bridging the patient-provider health information gap. Health Aff (Millwood).

[15] Ball Marion J, Smith Carla, Bakalar Richard S (2007). Personal health records: empowering consumers. J Healthc Inf Manag.

[16] Sequist Thomas D, Zaslavsky Alan M, Marshall Richard, Fletcher Robert H, Ayanian John Z (2009). Patient and physician reminders to promote colorectal cancer screening: a randomized controlled trial. Arch Intern Med.

[17] Pagliari Claudia, Detmer Don, Singleton Peter (2007). Potential of electronic personal health records. BMJ.

[18] Frost Jeana H, Massagli Michael P (2008). Social uses of personal health information within PatientsLikeMe, an online patient community: what can happen when patients have access to one another's data. J Med Internet Res.

[19] Kaelber David C, Jha Ashish K, Johnston Douglas, Middleton Blackford, Bates David W (2008). A research agenda for personal health records (PHRs). J Am Med Inform Assoc.

[20] DiMaggio P, Hargittai E, Neumann WR (2001). Social implications of the Internet. Annu Rev Sociol.

[21] Horrigan JB (2008). Home broadband adoption.

[22] Murdock G (2002). Tackling the digital divide: Evidence and intervention.

[23] Weingart Saul N, Rind David, Tofias Zachary, Sands Daniel Z (2006). Who uses the patient internet portal? The PatientSite experience. J Am Med Inform Assoc.

[24] Hsu John, Huang Jie, Kinsman James, Fireman Bruce, Miller Robert, Selby Joseph, Ortiz Eduardo (2005). Use of e-Health services between 1999 and 2002: a growing digital divide. J Am Med Inform Assoc.

[25] Kim E, Stolyar A, Lober WB, Herbaugh AL, Shinstrom SE, Zierler B, Soh C, Kim Y (2007). Usage patterns of a personal health record by elderly and disabled users. AMIA Annu Symp Proc.

[26] Lober W B, Zierler B, Herbaugh A, Shinstrom S E, Stolyar A, Kim E H, Kim Y (2006). Barriers to the use of a personal health record by an elderly population. AMIA Annu Symp Proc.

[27] Kling R (1999). Can the 'Next-Generation Internet' effectively support 'Ordinary Citizens'?. Inform Soc.

[28] Ralston James D, Hereford James, Carrell David (2006). Use and satisfaction of a patient Web portal with a shared medical record between patients and providers. AMIA Annu Symp Proc.

[29] Ralston James D, Carrell David, Reid Robert, Anderson Melissa, Moran Maureena, Hereford James (2007). Patient web services integrated with a shared medical record: patient use and satisfaction. J Am Med Inform Assoc.

[30] Keniston K (2004). Introduction: The four digital divides. Keniston K, Kumar D, editors. IT Experience in India.

[31] Tjora Aksel, Tran Trung, Faxvaag Arild (2005). Privacy vs usability: a qualitative exploration of patients' experiences with secure Internet communication with their general practitioner. J Med Internet Res.

[32] Wang Maisie, Lau Christopher, Matsen Frederick A, Kim Yongmin (2004). Personal health information management system and its application in referral management. IEEE Trans Inf Technol Biomed.

[33] Kushniruk A W, Patel C, Patel V L, Cimino J J (2001). 'Televaluation' of clinical information systems: an integrative approach to assessing Web-based systems. Int J Med Inform.

[34] Homer C S, Davis G K, Everitt L S (1999). The introduction of a woman-held record into a hospital antenatal clinic: the bring your own records study. Aust N Z J Obstet Gynaecol.

[35] Ross Stephen E, Lin Chen-Tan (2003). The effects of promoting patient access to medical records: a review. J Am Med Inform Assoc.

[36] Kim E, Mayani A, Modi S, Kim Y, Soh C (2005). Evaluation of patient-centered electronic health record to overcome digital divide. Conf Proc IEEE Eng Med Biol Soc.

[37] Follen Marilyn, Castaneda Rachel, Mikelson Melissa, Johnson Debrah, Wilson Alisa, Higuchi Keiko (2007). Implementing health information technology to improve the process of health care delivery: a case study. Dis Manag.

[38] Gustafson David H, McTavish Fiona M, Stengle William, Ballard Denise, Hawkins Robert, Shaw Bret R, Jones Ellen, Julèsberg Karen, McDowell Helene, Chen Wei Chih, Volrathongchai Kanittha, Landucci Gina (2005). Use and Impact of eHealth System by Low-income Women With Breast Cancer. J Health Commun.

[39] Gustafson David H, McTavish Fiona M, Stengle William, Ballard Denise, Jones Ellen, Julesberg Karen, McDowell Helene, Landucci Gina, Hawkins Robert (2005). Reducing the digital divide for low-income women with breast cancer: a feasibility study of a population-based intervention. J Health Commun.

[40] Trief Paula M, Sandberg Jonathan, Izquierdo Roberto, Morin Philip C, Shea Steven, Brittain Rebecca, Feldhousen Elizabeth Banks, Weinstock Ruth S (2008). Diabetes management assisted by telemedicine: patient perspectives. Telemed J E Health.

[41] Steinbrook Robert (2008). Personally controlled online health data--the next big thing in medical care?. N Engl J Med.

